# Calcium signaling in psoriasis: from pathogenesis to therapeutic opportunities

**DOI:** 10.3389/fimmu.2026.1881059

**Published:** 2026-07-15

**Authors:** Y. X. Chen, D. X. Zhuo, F. F. Wang

**Affiliations:** 1 Department of Dermatology, Shunyi Hospital of Beijing Traditional Chinese Medicine Hospital, Beijing, China; 2 Departments of Laboratory Medicine, Sanming First Hospital Affiliated to Fujian Medical University, Sanming, Fujian, China

**Keywords:** calcium signaling, dendritic cell, keratinocyte, NFAT, ORAI1, psoriasis, SOCE, STIM1

## Abstract

Psoriasis is a chronic inflammatory skin disease driven by the synergistic interplay between aberrant epidermal proliferation and immune imbalance, with its core pathological process centered on the IL-23/IL-17 inflammatory axis. In recent years, accumulating evidence has demonstrated that calcium (Ca^2+^) signaling not only participates in keratinocyte (KC) differentiation and the maintenance of skin barrier homeostasis but also serves as a critical upstream regulator of the maturation and activation of immune cells, including dendritic cells (DCs), Th17 cells, neutrophils, and mast cells. This review systematically summarizes the mechanistic roles of Ca^2+^ signaling dysregulation in psoriatic keratinocytes and multiple immune effector cells, with particular emphasis on the regulatory functions of SOCE within the DC–Th17 inflammatory axis. Current evidence indicates that the collapse of epidermal structural support systems, impaired Ca^2+^ sensing, and SOCE dysfunction collectively contribute to insufficient local Ca^2+^ signaling and defective KC differentiation. In contrast, persistently activated Ca^2+^-dependent signaling within the immune compartment promotes Th17 polarization, IL-17A release, and inflammatory cascade amplification through downstream pathways including Calcineurin–NFAT–RORγt. Based on these observations, this review further proposes that “compartment-specific bidirectional calcium dysregulation” may represent an underrecognized pathological pattern in psoriasis, characterized by the coexistence of impaired epidermal calcium signaling and persistent immune calcium hyperactivation. Furthermore, this review discusses therapeutic strategies targeting Ca^2+^ signaling and their translational challenges, including SOCE-targeted interventions and future combinatorial therapeutic approaches. Overall, this review reappraises the pathogenesis of psoriasis from the perspective of Ca^2+^ signaling with the aim of providing a novel theoretical basis for future precision immunomodulation and the development of innovative therapeutic strategies.

## 1 Introduction

### 1.1 Pathological features of psoriasis

Psoriasis is a chronic inflammatory disease primarily manifesting in the skin, with core pathological features involving several intertwined biological processes. Initially, keratinocytes (KCs) exhibit an imbalance between proliferation and differentiation: the cell proliferation cycle is significantly shortened, apoptosis is reduced, and the expression of terminal differentiation markers (e.g., loricrin and involucrin) is suppressed, leading to acanthosis, parakeratosis, and impaired skin barrier function (1–3). In parallel, the innate and adaptive immune systems undergo synergistic dysregulation: activated dendritic cells (DCs) secrete interleukin-23 (IL-23), driving the differentiation of CD4^+^ T cells into T helper 17 (Th17) cells and the production of effector cytokines; coupled with the infiltration of innate immune cells, such as neutrophils and macrophages, further amplifying the inflammatory response (4–7). Ultimately, the interleukin-23/interleukin-17 (IL-23/IL-17) inflammatory axis emerges as the key driver of the disease, in which IL-17A acts directly on keratinocytes to promote their proliferation, inhibit differentiation, and induce the expression of pro-inflammatory antimicrobial peptides and chemokines, thereby enhancing the recruitment and effector functions of immune cells (8–11). Collectively, these processes are mutually reinforced through a positive feedback mechanism: barrier impairment triggers the release of self-antigens and activates dendritic cells, IL-23 drives the IL-17 response, and IL-17 further exacerbates keratinocyte abnormalities, ultimately forming a self-sustaining pathological loop (12, 13).

### 1.2 Calcium signaling in psoriasis

Calcium ions (Ca²^+^) are among the most versatile and ubiquitous second messengers in eukaryotic biology, regulating a remarkable diversity of cellular processes including gene transcription, cell proliferation, differentiation, apoptosis, and immune activation (14–16). The specificity of calcium signaling is achieved through precise spatiotemporal control of cytosolic Ca^2+^ concentration, with cells employing a sophisticated toolkit of channels, pumps, and buffering proteins to generate signals ranging from local microdomains to global waves (16–18). In the context of psoriasis, Ca^2+^ ions exert dual regulatory roles. In epithelial tissues, Ca^2+^ regulates keratinocyte differentiation and maintains barrier homeostasis (3, 19), whereas in immune cells it drives cellular activation and amplifies the production of pro-inflammatory cytokines (12). This divergence reflects a high degree of context dependency, whereby the same Ca^2+^ signal elicits distinct functional outcomes across different cell types. Collectively, calcium signaling may function as an upstream integrative hub coordinating multiple inflammatory responses, thereby contributing to the initiation and progression of the disease (20, 21).

### 1.3 The SOCE pathway: importance and breadth in the psoriatic immune microenvironment

Store-operated calcium entry (SOCE), primarily mediated by the endoplasmic reticulum (ER) calcium sensor stromal interaction molecule 1 (STIM1) and the plasma membrane channel ORAI calcium release-activated calcium modulator 1 (ORAI1), represents the predominant Ca^2+^ influx mechanism in non-excitable cells (20, 22–24). Upon depletion of ER Ca^2+^ stores, STIM1 oligomerization activates ORAI1 channels to induce sustained Ca^2+^ entry, thereby triggering downstream pathways such as the calcineurin–nuclear factor of activated T cells (Calcineurin–NFAT) signaling cascade (21, 25). SOCE represents a central and broadly operative calcium influx mechanism in the psoriatic immune microenvironment, governing effector functions across both innate and adaptive immune cells. It orchestrates diverse cellular functions, ranging from Th17 cell differentiation and effector maintenance in T cells to dendritic cell maturation (4, 26), as well as chemotaxis in neutrophils and degranulation in mast cells (27, 28).The shared dependence of different immune cell types on SOCE suggests a possibility that dysregulated calcium homeostasis between cells may be a unifying yet underrecognized feature of psoriasis.

### 1.4 Core thesis and scope of this review

Accumulating evidence suggests the existence of an important yet not fully integrated feature in psoriasis pathogenesis — a compartment-specific bidirectional calcium dysregulation: attenuated calcium signaling in keratinocytes impairs differentiation, while persistently hyperactive calcium signaling in immune cells, particularly T lymphocytes, drives excessive effector polarization. In epidermal compartment, the loss of the physiological calcium gradient in keratinocytes, together with the downregulation of calcium signaling components, is associated with impaired differentiation and disruption of epidermal homeostasis (2, 29). In contrast, calcium-dependent signaling within the immune compartment, particularly SOCE-mediated signaling in T lymphocytes, remains persistently activated, thereby promoting pathogenic immune activation and pro-inflammatory cytokine production (10, 11, 21).

This review summarizes current evidence on calcium signaling dysregulation in major psoriatic effector cells and discusses its implications for disease pathogenesis and therapeutic intervention. Building on this foundation, we further evaluate therapeutic strategies targeting calcium signaling pathways, encompassing agents currently in clinical use as well as those under investigation, and propose a combinatorial intervention concept grounded in the principle of compartment-specific calcium rebalancing. By revisiting psoriasis through the lens of calcium signaling, this review seeks to offer complementary insights into its molecular basis and inform future research directions.

## 2 Keratinocyte-mediated maintenance of the epidermal calcium gradient and its dysregulation in psoriasis

**Figure 1:**
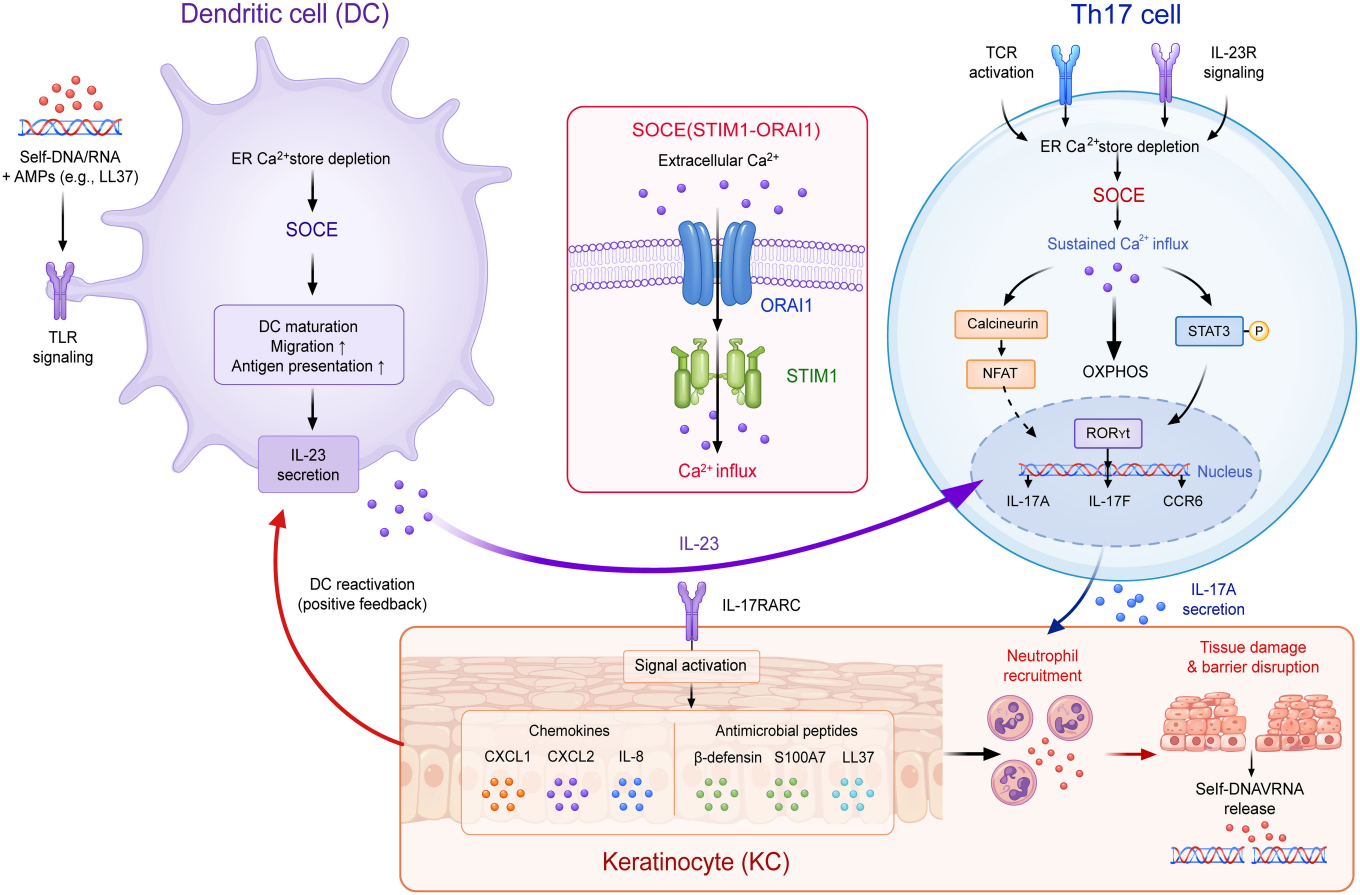
The Ca^2+^-driven DC-Th17-KC positive feedback loop in psoriasis: Calcium signaling, particularly via SOCE, drives DC maturation and IL-23/IL-17 axis activation. Th17-derived cytokines stimulate keratinocytes (KCs) to release chemokines and AMPs, promoting neutrophil recruitment and further immune cell infiltration. This creates a self-amplifying positive feedback loop that perpetuates chronic skin inflammation and epidermal hyperplasia.

In normal epidermis, the Ca^2+^ concentration gradient that progressively increases from the basal layer to the stratum granulosum serves as the core physiological foundation for maintaining KC terminal differentiation and skin barrier homeostasis (30, 31). In psoriasis, the intrinsic programs governing KC proliferation, differentiation, and Ca^2+^ homeostasis undergo triple systemic dysregulation, ultimately leading to collapse of the epidermal Ca^2+^ gradient and further driving abnormal proliferation and inflammatory microenvironment formation (12, 32) (**Figure 1**).

### 2.1 Maintenance and imbalance of the physical barrier

Under physiological conditions, the epidermal Ca^2+^ gradient primarily relies on the physical barrier constructed by KCs. The stratum corneum is essentially the product of KC terminal differentiation: basal KCs continuously proliferate and migrate upward, differentiating within the stratum granulosum into keratin-rich corneocytes. Simultaneously, they secrete lamellar bodies, which release lipids, enzymes, and antimicrobial peptides to form a dense extracellular lipid lamellar structure (19, 33). This corneal barrier, established through KC differentiation and secretory activities, effectively restricts transepidermal water loss (TEWL) and outward Ca^2+^ diffusion, thereby maintaining a localized high-calcium environment within the stratum granulosum. In addition, tight junctions (TJs) located in the stratum granulosum are synthesized and anchored by KCs, further limiting paracellular Ca^2+^ diffusion and stabilizing local calcium peaks (19, 33).

However, in psoriasis, the biological behavior of KCs undergoes profound alterations. Abnormal proliferation and impaired terminal differentiation result in disorganized stratum corneum architecture, defective lamellar body secretion, and disturbed lipid organization, thereby directly compromising barrier integrity (2, 34). Concurrently, dysregulated expression of TJ-associated proteins, such as Claudins, further reduces the Ca^2+^ retention capacity of the stratum granulosum (35). As KCs fail to generate a structurally intact and functionally competent stratum corneum, Ca^2+^ is continuously lost alongside increased TEWL, leading to progressive collapse of the high-calcium microenvironment normally maintained within the upper epidermis (30, 31). Collectively, these findings indicate that KCs are not only builders of the epidermal barrier, but also direct determinants of epidermal Ca^2+^ gradient maintenance.

### 2.2 Maintenance and imbalance of calcium sensing and signal transduction (active regulation)

Beyond their structural role, KCs also function as the principal sensing and processing units of epidermal Ca^2+^ signaling. Under normal conditions, the calcium-sensing receptor (CaSR) expressed on the KC membrane dynamically detects extracellular Ca^2+^ fluctuations and activates the PLC–IP_3_ signaling pathway, thereby inducing Ca^2+^ release from the ER (2, 33). As the major intracellular Ca^2+^ reservoir in KCs, the ER further amplifies Ca^2+^ signaling and drives expression of terminal differentiation-associated genes, including KRT1, KRT10, and Filaggrin (19, 33). Recent studies further suggest that the high-calcium environment within the stratum granulosum largely originates from intracellular calcium storage systems, indicating that the ER is not only a signaling amplifier but also an integral component of the epidermal Ca^2+^ gradient itself (36). In addition, KC-derived regulatory proteins, such as SerpinB7, participate in maintaining ER calcium homeostasis and Ca^2+^ influx responses (3).

In psoriasis, however, the ability of KCs to sense and process Ca^2+^ signals is markedly impaired. Studies have demonstrated that CaSR expression is significantly downregulated in psoriatic KCs, rendering these cells unable to effectively initiate differentiation-related signaling pathways even under high-calcium conditions (2). Meanwhile, abnormalities in SerpinB7 further weaken Ca^2+^ influx and destabilize ER calcium stores, preventing Ca^2+^ signals from being properly translated into differentiation cues (3). As dysfunction of the CaSR–ER axis progresses, KCs gradually lose their ability to appropriately respond to local Ca^2+^ environments and eventually remain trapped in a state characterized by low differentiation and persistent inflammation (3).

### 2.3 Maintenance and imbalance of calcium store replenishment and dynamic balance (passive supply)

Long-term Ca^2+^ homeostasis further depends on intrinsic KC-mediated calcium replenishment and dynamic balancing systems. Among these, SOCE represents the central regulatory mechanism. Upon ER calcium depletion, STIM1 located on the ER membrane senses the decline in ER Ca^2+^ levels and subsequently activates ORAI1/ORAI3 channels on the plasma membrane, consequently mediating sustained extracellular Ca^2+^ influx to refill ER calcium stores (12, 29). In parallel, KCs express multiple Ca^2+^ channels, including TRPC1, TRPC4, TRPC6, and TRPV6, which collectively establish a multilayered Ca^2+^ replenishment network (1). These systems enable KCs to autonomously maintain intracellular Ca^2+^ homeostasis and ensure continuity of the differentiation process.

In psoriasis, the intrinsic Ca^2+^ replenishment machinery of KCs undergoes systemic failure. Expression of STIM1, ORAI1, and ORAI3 is markedly decreased, resulting in impaired SOCE activity and insufficient ER calcium refilling (37). This downregulation of SOCE in KCs consequently leads to insufficient calcium signaling required for normal differentiation and homeostatic maintenance (12). Similarly, expression of TRPC family members and TRPV6 is broadly downregulated, further restricting transmembrane Ca^2+^ influx (1, 2). Importantly, these Ca^2+^ influx defects persist even after psoriatic KCs are removed from inflammatory microenvironments and serially passaged *in vitro*, suggesting that these abnormalities represent intrinsic KC defects rather than secondary consequences of external inflammation (1, 38). Due to chronic dysfunction of SOCE and associated Ca^2+^ channel systems, psoriatic KCs consistently fail to generate adequate Ca^2+^ signaling responses, eventually remaining in an abnormal proliferative state resembling persistent low-calcium conditions, which further promotes epidermal hyperplasia and chronic inflammation (12).

## 3 SOCE-driven immune activation: the DC–T cell axis in psoriasis

### 3.1 SOCE-dependent calcium signaling in dendritic cells

Dendritic cells (DCs) are professional antigen-presenting cells that serve as pivotal sentinels bridging innate and adaptive immunity (39, 40). Under steady-state conditions in the skin, various DC subsets such as Langerhans cells and dermal DCs continuously patrol peripheral tissues to maintain immune tolerance (41). However, this equilibrium is disrupted in psoriatic lesions: keratinocyte-derived antimicrobial peptides form complexes with self-DNA/RNA, which in turn activate DCs via Toll-like receptor (TLR) signaling, triggering an inflammatory cascade (42, 43).

Growing evidence identifies calcium signaling as a master switch for DC maturation, migration, and antigen presentation (40, 44, 45). Specifically, the SOCE plays a decisive role in DC functionality. Studies have demonstrated that Stim1 deficiency leads to an approximately 70% reduction in SOCE and significantly impairs the formation of localized calcium microdomains. This deficit in calcium signaling markedly diminishes DC chemotactic responsiveness to gradients such as SDF-1 and obstructs phagosome-lysosome fusion, thereby mechanistically compromising the efficiency of antigen cross-presentation (45).

In DCs, calcium influx triggers the calcineurin-dependent nuclear translocation of NFAT, which modulates terminal differentiation, apoptosis, and cytokine expression (46). Given that DCs are the primary cellular source of IL-23 in psoriatic lesions (41, 43, 47), it is plausible to speculate that aberrant activation of the SOCE–calcineurin–NFAT axis may drive the overproduction and secretion of IL-23. However, direct evidence supporting this regulatory mechanism within the psoriatic microenvironment remains limited. One possible explanation is the marked heterogeneity of dendritic cell populations in psoriasis, including conventional dendritic cells, inflammatory monocyte-derived dendritic cells, and plasmacytoid dendritic cells, which may exhibit distinct calcium signaling dependencies and cytokine programs (40, 41). This heterogeneity poses a significant challenge for mechanistic studies, as distinct DC subsets may differ substantially in their capacity to produce IL-23 and in their dependence on SOCE signaling, making it difficult to determine whether activation of the SOCE–calcineurin–NFAT axis directly regulates IL-23 expression in the relevant pathogenic DC populations.

### 3.2 SOCE-dependent calcium signaling in Th17 cell differentiation and pathogenicity

The SOCE serves as the central molecular engine driving Th17 cell polarization and pathogenic differentiation. Accumulating evidence demonstrates that SOCE-mediated sustained Ca^2+^ signaling is indispensable for maintaining Th17-associated transcriptional programs and effector functions, and its aberrant activation is closely implicated in the pathological inflammatory responses characteristic of psoriasis (21, 48, 49).

A key downstream mechanism of SOCE is the activation of the Calcineurin–NFAT signaling axis. Sustained Ca^2+^ influx activates the phosphatase calcineurin, which dephosphorylates NFAT and facilitates its nuclear translocation (26, 50, 51). Once in the nucleus, NFAT cooperates with transcription factors such as STAT3 to directly promote the expression of Th17 lineage-defining transcription factors, including RORγt and RORα, thereby driving Th17 cell differentiation (52, 53). Beyond transcriptional regulation, emerging evidence indicates that SOCE also participates in maintaining the metabolic homeostasis and effector functions of pathogenic Th17 cells. STIM1-mediated calcium signaling is essential for preserving mitochondrial electron transport chain integrity and oxidative phosphorylation (OXPHOS) activity (48). In pathogenic Th17 cells lacking STIM1, OXPHOS function is markedly impaired, accompanied by aberrant accumulation of reactive oxygen species (ROS), which subsequently causes DNA damage and compromises cell survival. Conversely, direct inhibition of OXPHOS redirects pathogenic Th17 cells toward a non-pathogenic gene expression profile (48). Thus, SOCE not only determines early Th17 cell differentiation through the NFAT–RORγt axis but also sustains their pathogenic effector functions through regulation of mitochondrial metabolism.

### 3.3 SOCE coordinates the DC–Th17 inflammatory circuit in psoriasis

Within the inflammatory microenvironment of psoriasis, a highly coupled immune amplification loop is established between dendritic cells (DCs) and pathogenic Th17 cells, with SOCE serving as the central calcium signaling hub linking these processes (48, 49). Activated DCs drive Th17 cell polarization by secreting IL-23 while simultaneously enhancing T-cell receptor (TCR)-dependent activation signals. Subsequently, sustained Ca^2+^ influx mediated by SOCE activates the calcineurin–NFAT pathway (26, 50, 51), which synergizes with IL-23-induced STAT3 signaling to promote the expression of key pathogenic genes such as RORC (encoding RORγt) and IL17A, thus sustaining the pathogenic effector functions of Th17 cells (52–54).

**Figure 2:**
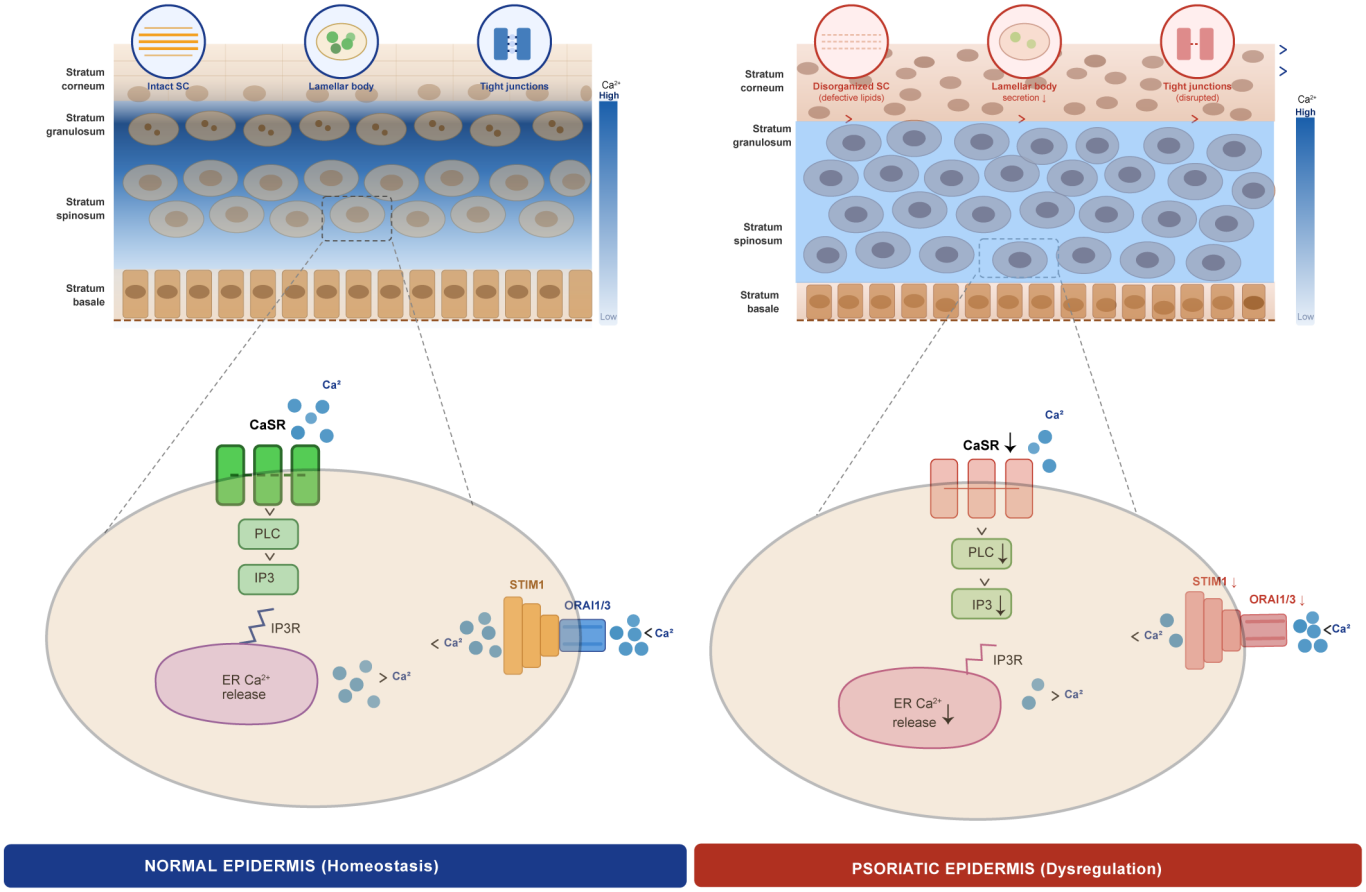
Normal skin (left) displays a distinct epidermal calcium gradient, normal keratinocyte differentiation and calcium signal transduction; psoriatic lesions (right) show the loss of the calcium gradient, parakeratosis, and downregulation of multiple calcium channel proteins.

Following this activation, IL-17A released by Th17 cells acts on keratinocytes to induce the expression of chemokines and antimicrobial peptides, promoting neutrophil recruitment and epidermal hyperproliferation (13). These inflammatory responses continuously reactivate DCs and form a self-amplifying positive feedback loop of chronic inflammation (10). SOCE not only participates in T-cell differentiation but likely serves as a critical integration platform connecting DC-derived cytokine signals with endogenous T-cell activation programs. Consequently, the DC → IL-23 → Th17 → IL-17A axis represents a core mechanism for the persistence of chronic inflammation in psoriasis, providing a novel theoretical foundation for SOCE-targeted therapeutic interventions (21, 55) (**Figure 2**).

## 4 Calcium signaling in other psoriatic effector cells

Beyond keratinocytes, T cells, and dendritic cells, the psoriatic immune microenvironment comprises various other effector cells that play critical roles in disease progression. While calcium signaling is likely a fundamental driver of their activation and specialized functions, direct evidence and dedicated literature regarding these specific cell types remain relatively sparse. Consequently, this chapter consolidates the existing research on these additional effector cells to provide a comprehensive overview of the calcium-dependent regulatory network in psoriasis.

### 4.1 Calcium-dependent regulation of macrophages in psoriasis

Under normal physiological conditions, skin-resident macrophages maintain tissue homeostasis and limit excessive inflammatory responses by rapidly producing the anti-inflammatory cytokine IL-10 via the TLR2/4–p38/ERK–CREB signaling pathway (56, 57). In psoriasis, macrophages polarize toward a pro-inflammatory M1 phenotype characterized by high expression of TLR7–9 and various inflammatory mediators, while anti-inflammatory M2 macrophages decrease, which eventually leads to the gradual failure of the negative feedback regulation required for inflammatory resolution and the destruction of this immune homeostasis (58, 59).

Recent studies have further identified calcium/calmodulin-dependent protein kinase IV (CaMK4), which is driven by calcium signaling, as a critical molecular driver of this pathological transition. CaMK4 is markedly upregulated in both human psoriatic lesions and imiquimod (IMQ)-induced murine models (34). Mechanistically, CaMK4 directly interacts with adenylate cyclase 1 (ADCY1) and inhibits the ADCY1–cAMP signaling axis, which subsequently reduces the phosphorylation levels of ERK and p38, ultimately suppressing the transcription and translation of IL-10. This reduction in IL-10 weakens the anti-inflammatory “braking” mechanism in psoriatic skin, thereby facilitating the persistence of chronic inflammation. Conversely, in CaMK4-deficient macrophages, ADCY1 expression and cAMP signaling are restored, leading to enhanced activation of ERK1/2 and p38 and a significant increase in IL-10 production, which is accompanied by a marked alleviation of psoriatic inflammation (34).

In conclusion, calcium signaling plays a pivotal role in macrophage polarization and the negative feedback regulation of inflammation. Its dysregulation represents a key mechanism underlying the persistence of chronic inflammation in psoriasis. These findings further suggest that targeting CaMK4 and its associated calcium signaling pathways may serve as a promising therapeutic strategy for clinical intervention in psoriasis.

### 4.2 Calcium-dependent regulation of neutrophil in psoriasis

Under normal physiological conditions, neutrophils serve as the primary defense line of the skin’s innate immunity, executing a highly precise “recognition–response–resolution” program. Guided by chemokine gradients from keratinocytes, neutrophils rapidly migrate to infection sites to eliminate pathogens through phagocytosis, degranulation, and the formation of neutrophil extracellular traps (NETs) (60, 61). Subsequently, neutrophils promptly enter apoptosis and are cleared by macrophages, promoting the timely resolution of inflammation (62). Notably, this entire life cycle is under strict spatiotemporal control by calcium signaling. Specifically, pulsed Ca^2+^ elevations generated by STIM1–ORAI1-mediated SOCE are essential for neutrophil chemotaxis and antimicrobial functions, while the rapid restoration of Ca^2+^ to resting levels facilitates the termination of inflammation (28 ,63).

In psoriasis, this sophisticated regulatory program is significantly remodeled by the inflammatory microenvironment. Keratinocytes continuously produce chemokines such as CXCL1, CXCL2, and IL-8 via IL-1R1-dependent pathways, which combined with activated vascular endothelial signals, create sustained chemotactic gradients that promote aberrant neutrophil recruitment (12, 64). In this inflammatory milieu, the STIM1–SOCE pathway is persistently activated, maintaining LFA-1 in a high-affinity state and enhancing transendothelial migration through ORAI1-mediated local Ca^2+^ influx (28, 65). This “calcium-locked” state allows neutrophils to bypass normal spatiotemporal regulation and continuously infiltrate the epidermis, ultimately forming Munro’s microabscesses (28, 64).

Furthermore, the infiltrated neutrophils do not enter the normal apoptotic program but instead exhibit delayed apoptosis and abnormal NETosis. Sustained Ca^2+^ influx activates the PKC–ROS–PAD4/NE signaling axis, driving the massive release of NETs containing LL37–DNA complexes (61, 66). These NETs further amplify psoriatic inflammation through multiple mechanisms, including the activation of plasmacytoid dendritic cells (pDCs) via TLR9 and the cGAS–STING pathways to induce type I interferon production (61, 67), as well as the enhancement of Th17 cell differentiation through Act1/TRAF3IP2 signaling (61). Ultimately, this establishes a self-amplifying inflammatory loop—”NETs → Th17 → keratinocyte activation → chemokine release → increased neutrophil recruitment”—thereby sustaining chronic inflammation in psoriasis.

### 4.3 Calcium-dependent regulation of mast cell in psoriasis

Under physiological homeostasis, mast cells (MCs) typically execute a transient and stimulus-coupled activation program. Upon recognition of allergens, pathogens, or damage-associated signals, calcium signaling is initiated by IP_3_ receptor-mediated release from the ER (68, 69), which is subsequently amplified by STIM1–ORAI1-dependent SOCE to trigger controlled degranulation, eicosanoid metabolism, and cytokine secretion (70). During this process, MCs rapidly release pre-stored inflammatory mediators such as histamine, tryptase, and TNF-α, while simultaneously inducing the production of lipid mediators like prostaglandin D_2_ (PGD_2_) and leukotrienes (27, 68, 71). Furthermore, they promote the expression of cytokines including IL-6, IL-17A, and GM-CSF to facilitate local immune defense and tissue repair. Subsequently, as Ca^2+^ homeostasis is restored, MCs re-enter a resting state and gradually replenish their granular contents, thereby preventing sustained inflammatory damage (68).

Research indicates a significant increase in the number of activated MCs within psoriatic lesions, primarily aggregating around dermal blood vessels and the dermo-epidermal junction (27). Notably, these activated MCs persist even in clinically resolved lesions, suggesting their potential involvement in disease recurrence and the maintenance of inflammatory memory (71). In psoriatic lesions meeting clinical remission standards, MCs have been confirmed as the source of over 95% of IL-17-positive cells. In addition to IL-17A, aberrantly activated MCs continuously release TNF-α, IL-6, VEGF, and various chemokines, thereby promoting angiogenesis, neutrophil recruitment, and abnormal keratinocyte proliferation, which further amplifies the local inflammatory response (27).

Current evidence suggests that the persistent activation of mast cells in resolved psoriatic lesions may be sustained by continuous cytokine stimulation from the local inflammatory niche, particularly through interactions with tissue-resident memory T (T_RM) cells (27, 71). Specifically, MCs remain in close proximity to CD103^+^ T_RM cells that persist after clinical resolution, and bidirectional cytokine-mediated crosstalk between these cell populations may repeatedly provide stimulatory signals to MCs (71, 72). Such interactions could lead to recurrent SOCE activation, thereby maintaining a calcium-dependent pro-inflammatory state without requiring continuous autonomous calcium store depletion. This process may contribute to the sustained production of inflammatory mediators and support the persistence of IL-17A-positive MCs within psoriatic lesions.

Although mechanistic evidence directly linking calcium signaling dysregulation to psoriatic MC dysfunction remains limited, the well-established role of SOCE in regulating MC degranulation and cytokine secretion (68, 73) suggests that persistent abnormalities in calcium signaling may critically contribute to the pro-inflammatory activation of MCs.

## 5 Therapeutic targeting of calcium signaling in psoriasis

### 5.1 Restoring epidermal calcium homeostasis

Current therapeutic strategies targeting epidermal calcium homeostasis disruption in psoriasis are primarily centered on the application of vitamin D and its analogues. Calcipotriol, calcitriol, and alfacalcidol all belong to the vitamin D pharmacological family: calcitriol represents the drug form of active vitamin D [1,25(OH)_2_D_3_], calcipotriol is a structural analogue thereof, and alfacalcidol is a prodrug derivative that undergoes *in vivo* conversion to active vitamin D. Mechanistically, active vitamin D activates the vitamin D receptor (VDR) to modulate intracellular Ca^2+^ signaling, promotes the expression of terminal differentiation-associated proteins such as filaggrin and loricrin, and concurrently suppresses aberrant keratinocyte proliferation (74–76). These actions are highly relevant to the pathological changes observed in psoriasis, particularly the collapse of the epidermal calcium gradient and the impairment of keratinocyte differentiation (74).

Additionally, VDR signaling is not confined to the regulation of keratinocyte differentiation but may further modulate the Th17/Treg immune balance, suggesting that restoration of epidermal calcium homeostasis may confer concurrent immunomodulatory benefits (76). Accordingly, the therapeutic significance of vitamin D analogues extends beyond the correction of keratinization abnormalities; they may be conceptualized as a calcium homeostasis reconstitution strategy specifically targeting the impaired epidermal calcium signaling characteristic of psoriasis (75).

### 5.2 Direct SOCE-targeting strategies

As the central role of SOCE in the psoriatic immune microenvironment has become increasingly established, interventional strategies directly targeting the STIM–ORAI axis have attracted considerable attention. Notably, in keratinocytes, STIM1–ORAI1 signaling primarily contributes to gradual calcium store refilling and supports calcium gradient–driven differentiation programs, processes that are less critically dependent on sustained SOCE activity than immune-cell activation (19, 29). In contrast, T cells rely on SOCE to generate the sustained and non-redundant Ca^2+^ signals required for NFAT nuclear translocation and activation-induced cytokine transcription. Attenuated calcium influx is often insufficient to maintain these transcriptional programs, reflecting a greater functional dependence on SOCE in immune cells than in keratinocytes (26, 51). This asymmetry suggests that SOCE inhibition may preferentially restrain pathogenic T-cell activation while exerting comparatively milder effects on keratinocyte homeostasis, thereby supporting the rationale for targeting the SOCE pathway as a therapeutic strategy in psoriasis. By inhibiting Ca^2+^ influx at its source, SOCE inhibitors act at a step upstream of the inflammatory cascade, with the potential to fundamentally block the subsequent activation of Calcineurin–NFAT signaling and Th17-associated inflammatory programs (77–79).

Several SOCE-targeting agents have recently demonstrated therapeutic potential in psoriasis-related experimental models and early clinical studies. Among them, BTP2 (YM-58483) suppresses ORAI1-mediated Ca^2+^ influx and inhibits keratinocyte proliferation in HaCaT cells as an extensively characterized CRAC/SOCE inhibitor (49, 79). Celastrol exerts dual inhibitory effects on STIM1 clustering and ORAI1 activation to disrupt STIM1–ORAI1 coupling and suppress SOCE activation. This compound significantly ameliorated skin inflammation and pathological changes in the imiquimod-induced psoriasis mouse model (49). In addition to preclinical compounds, several CRAC channel inhibitors have entered clinical evaluation for psoriasis. CM2489 was the first CRAC channel blocker tested in humans for moderate-to-severe plaque psoriasis and completed phase I clinical trials. However, its development was subsequently discontinued because of limited efficacy (78–80). The selective ORAI1 inhibitor PRCL-02 has recently progressed to phase IIa clinical trials for chronic plaque psoriasis to further support the translational potential of SOCE-targeted therapeutic strategies (78).

These findings consolidate SOCE as a master immunoregulatory node in psoriasis, underscoring its potential as a prime therapeutic target. Although an increasing number of SOCE inhibitors have recently been identified, including natural compounds, synthetic small molecules, and even previously approved drugs with newly recognized SOCE-inhibitory properties (49, 81), translational therapeutic studies in psoriasis-specific models remain relatively limited. Further investigation is therefore warranted to better evaluate the therapeutic applicability, selectivity, and clinical potential of SOCE-targeted interventions in psoriasis.

### 5.3 Targeting calcium signal transduction

Beyond directly inhibiting SOCE-mediated Ca^2+^ influx, calcineurin inhibitors, which are widely employed in current clinical practice, exert their immunosuppressive effects primarily by blocking downstream calcium signal transduction. Cyclosporine A (CsA) forms a complex with cyclophilin, whereas tacrolimus (FK506) binds to FKBP12; both complexes specifically inhibit calcineurin activity, thereby blocking the Calcineurin–NFAT signaling axis (82, 83). Given that calcineurin activation is fundamentally dependent on SOCE-mediated Ca^2+^ signals (25, 84), these agents may be conceptualized as therapeutic strategies that “indirectly target the downstream effectors of SOCE.”

In clinical practice, CsA remains an important systemic treatment option for moderate-to-severe psoriasis (85, 86), while tacrolimus is primarily employed as a topical agent for sensitive anatomical sites such as the face and genitalia (85, 86). However, the long-term use of calcineurin inhibitors remains limited by systemic adverse effects such as nephrotoxicity and neurotoxicity (82, 87), largely because calcineurin is a ubiquitously expressed phosphatase essential for diverse physiological processes beyond the immune system (88). These limitations suggest that broadly suppressing this downstream decoder lacks the cell-type specificity required for precise therapy. As SOCE mediated by Orai1 represents a highly specialized calcium entry route within the immune system, targeting this upstream pathway could achieve more precise immunomodulation while minimizing systemic toxicity.

### 5.4 Summary of therapeutic strategies

The representative therapeutic agents targeting calcium signaling pathways in psoriasis, together with their primary targets,pharmacological effects, and current stages of clinical development, are summarized in **Table 1**.

**Table 1:** Summary of therapeutic agents targeting calcium signaling pathways in psoriasis.

Drug/agent	Drug class	Primary target	Primary pharmacological effects	Evidence level	Key references
Calcipotriol, Calcitriol, Alfacalcidol	Vitamin D analogues and derivatives	Vitamin D Receptor (VDR)	Activates VDR, modulates intracellular Ca^2+^ homeostasis, promotes keratinocyte terminal differentiation, and suppresses keratinocyte proliferation	Clinical application	(74–76) Barrea et al., 2017; Mai et al., 2025; Li and Chan, 2025
Celastrol	Natural SOCE modulator	STIM1, ORAI1	Prevents STIM1 puncta formation and directly inhibits ORAI1 activation, thereby disrupting STIM1–ORAI1 coupling and suppressing SOCE	Preclinical validation (HaCaT cells and IMQ-induced psoriasis mouse model)	(49) Yuan et al., 2023
BTP2 (YM-58483)	CRAC/SOCE inhibitor	ORAI1	suppresses ORAI1-mediated Ca^2+^ influx and T-cell activation, and inhibits keratinocyte proliferation by blocking SOCE	Preclinical validation (HaCaT cells, immune cell models, and animal inflammatory disease models)	(79, 81) Rubaiy, 2023; Yuan et al., 2023; Rahman and Rahman, 2017
CM2489	CRAC/SOCE inhibitor	ORAI1	Directly blocks ORAI1-mediated Ca^2+^ influx and downstream immune activation	Clinical trial	(78, 79) Rubaiy, 2023; Masson et al., 2021
PRCL-02	Selective ORAI1 inhibitor	ORAI1	Directly inhibits ORAI1-mediated Ca^2+^ influx and downstream Calcineurin–NFAT signaling	Clinical trial	(78) Masson et al., 2021
Cyclosporine A (CsA)	Calcineurin inhibitor	Cyclophilin–Calcineurin complex	Inhibits calcineurin phosphatase activity and blocks NFAT dephosphorylation and nuclear translocation	Clinical application	(82, 86) Lee et al., 2023; Elsisi et al., 2025
Tacrolimus (FK506)	Calcineurin inhibitor	FKBP12–Calcineurin complex	Suppresses Calcineurin–NFAT signaling and inhibits T-cell activation	Clinical application	(82, 86) Lee et al., 2023; Elsisi et al., 2025

## 6 Challenges and future perspectives

### 6.1 Future research directions

Despite substantial progress in understanding calcium signaling in psoriasis, several important questions remain unresolved.

The precise mechanisms by which SOCE–Calcineurin–NFAT signaling regulates IL-23 production in distinct dendritic-cell subsets within the psoriatic microenvironment require further investigation. Future studies employing dendritic cell-specific Stim1 or Orai1 conditional knockout mice, together with subset-specific analyses and single-cell transcriptomic approaches, may help clarify whether SOCE-dependent NFAT activation directly controls IL-23 production in psoriasis.

The role of SOCE in maintaining the persistent activation of IL-17A-positive mast cells also remains largely speculative. Future studies should focus on defining the functional interactions between mast cells and T_RM cells within psoriatic lesions, particularly whether recurrent cytokine-mediated stimulation from T_RM cells drives repeated SOCE activation and sustains mast-cell inflammatory memory.

Another important challenge concerns the quantitative characterization of epidermal calcium homeostasis. Although disruption of epidermal calcium homeostasis is a well-recognized feature of psoriasis, reliable quantitative measurements of calcium concentrations across different epidermal layers in healthy and psoriatic skin remain lacking (1, 19). Establishing such quantitative calcium maps may provide an important foundation for validating the concept of compartment-specific calcium dysregulation and guiding the development of future compartment-specific combination therapies aimed at simultaneously restoring epidermal calcium homeostasis while suppressing pathogenic immune activation.

### 6.2 Compartment-specific combination therapy

Growing evidence suggests that psoriasis may involve a “compartment-specific bidirectional calcium dysregulation,” in which the impaired epidermal calcium signaling and the persistent immune calcium hyperactivation together constitute a fundamental basis for disease initiation and maintenance. Within this framework, integrated therapeutic strategies based on compartment-specific calcium homeostasis reconstitution may provide a novel theoretical direction for psoriasis treatment.

In the epidermal compartment, vitamin D analogues activate VDR signaling to promote terminal differentiation of KCs, restore skin barrier integrity, thereby correcting the impaired epidermal calcium signaling (2, 33). Conversely, in the immune compartment, persistently activated SOCE mediates abnormal Ca^2+^ influx, driving Th17 polarization and amplification of chronic inflammation (22, 89). Accordingly, SOCE inhibitors may suppress the hyperactive calcium signaling state within the immune compartment and thereby block Calcineurin–NFAT- and Th17-associated inflammatory programs at their upstream source (89).

Importantly, the combination of these two strategies does not merely represent simple drug addition but rather targets the two key pathological compartments within the bidirectional calcium imbalance model, enabling mechanistic synergy. On the one hand, restoration of epidermal barrier integrity may reduce autoantigen exposure and dendritic cell activation. On the other hand, inhibition of immune Ca^2+^ signaling may alleviate persistent inflammatory stimulation of KCs, thereby jointly disrupting the vicious cycle of “barrier damage–immune activation (49, 85).”

Furthermore, this framework may complement existing biologic therapies. Current biologics, such as IL-17 inhibitors, primarily target downstream inflammatory cytokines, whereas calcium signaling rebalancing strategies may simultaneously restore epidermal calcium homeostasis and suppress aberrant immune Ca^2+^ signaling at a more upstream level (90–92). Therefore, future combinatorial approaches integrating SOCE-targeted therapies with vitamin D analogues, or even IL-17 inhibitors, may ultimately achieve more durable and precise disease control.

### 6.3 Challenges in SOCE-targeted therapy

Although SOCE inhibitors demonstrate significant potential in managing autoimmune inflammation, their clinical translation faces critical challenges. Within the “compartment-specific bidirectional calcium dysregulation” framework proposed in this article, psoriasis is characterized not only by persistent immune Ca^2+^ hyperactivity but also by epidermal calcium signaling insufficiency. While the SOCE pathway is predominantly expressed and functionally essential in immune cells, STIM1/ORAI1 proteins are also present in keratinocytes and other cell types, where they participate in maintaining baseline physiological functions (29, 93). Consequently, non-selective systemic SOCE inhibition may exert modest interference with Ca^2+^ homeostasis in non-target tissues while suppressing immune inflammation, thereby potentially exacerbating the epidermal “low-calcium” state.

The clinical experience with CM2489, the first CRAC channel inhibitor evaluated in psoriasis, further illustrates these translational challenges. Although the detailed reasons for its discontinuation have not been fully disclosed, CM2489 demonstrated only limited clinical efficacy in psoriasis (80). Pharmacokinetic factors, insufficient target engagement, and other biological barriers may all represent potential contributors to its discontinuation. Within the framework proposed in this review, it is also conceivable that non-selective systemic SOCE inhibition may have limited therapeutic efficacy by simultaneously affecting calcium-dependent physiological processes in non-target cell populations. Although this possibility remains speculative, the CM2489 experience underscores the importance of developing more selective and tissue-targeted SOCE-modulating strategies.

Accordingly, the pivotal question for future research may not simply be whether SOCE should be inhibited, but rather in which cells and by what means SOCE should be modulated. Through immune-cell-specific delivery strategies, such as nanoparticle-based systems designed to preferentially target dendritic cells or pathogenic T-cell populations, or through skin-localized exposure approaches including topical formulations and microneedle-assisted delivery platforms, future interventions may selectively suppress aberrant immune Ca^2+^ signaling while minimizing interference with epidermal calcium homeostasis, thereby ultimately enabling more precise compartment-specific calcium regulation.
